# Data-Driven Enhancements: Unleashing the Power of Data Science in Plastic Surgery

**DOI:** 10.7759/cureus.42357

**Published:** 2023-07-24

**Authors:** Mohd Altaf Mir

**Affiliations:** 1 Burns and Plastic Surgery, All India Institute of Medical Sciences, Bathinda, IND

**Keywords:** machin learning, computational modelling, surgical research, innovative teaching learning, clinical data science

## Abstract

Plastic surgery is a specialized field of medicine that focuses on reconstructing, restoring, or enhancing a person's appearance through surgical procedures. Over the years, data science has become increasingly relevant in this domain, offering valuable insights and innovations that can improve patient outcomes, enhance surgical techniques, and drive advancements in the field. Data science has revolutionized the field of plastic surgery by providing valuable insights, predictive models, and innovative tools that enhance surgical planning, patient outcomes, and research advancements.

## Editorial

Let us explore the role of data science in plastic surgery and its impact on both patients and surgeons.

Preoperative planning and decision-making

Data science can aid plastic surgeons in preoperative planning by leveraging historical patient data and computational modeling. By analyzing large datasets of patient information, such as medical records, imaging data (e.g., CT scans, MRI), and surgical outcomes, data scientists can develop predictive models to assist surgeons in decision-making. These models can help estimate surgical risks, predict postoperative complications, and optimize treatment plans tailored to each patient's unique characteristics [1-3].

Computer-assisted surgical simulation

The techniques of data science, combined with computer graphics and virtual reality, enable surgeons to simulate surgical procedures before performing them on patients. By creating realistic 3D models based on patient scans, surgeons can use data-driven simulations to visualize and plan surgeries, assess potential outcomes, and make informed decisions about the surgical approach. This technology enhances surgical precision, reduces complications, and improves patient safety [2-4].

Image analysis and facial recognition

Data science plays a crucial role in analyzing facial images and developing facial recognition algorithms that can aid plastic surgeons in facial reconstruction and aesthetic procedures. By utilizing machine learning techniques, data scientists can extract facial landmarks, measure facial symmetry, and analyze facial features. These analyses provide quantitative measurements and visualizations that assist surgeons in accurately assessing and planning facial surgeries, such as rhinoplasty, facelifts, and reconstructive procedures [3-5].

Patient outcome prediction

This science can help predict patient outcomes and optimize treatment plans by leveraging large-scale patient datasets and machine-learning algorithms. By analyzing factors, such as patient demographics, medical history, and surgical procedures, data scientists can develop predictive models that estimate the likelihood of successful surgical outcomes or identify patients at higher risk of complications. This information allows surgeons to personalize treatment approaches, set realistic expectations, and optimize postoperative care [1-5].

Postoperative monitoring and analysis

Data science techniques can enable continuous monitoring and analysis of postoperative patient data to detect complications, assess recovery progress, and optimize follow-up care. By integrating wearable devices, sensor data, and patient-reported outcomes, data scientists can develop algorithms that identify early signs of complications, provide real-time feedback to surgeons, and support timely interventions. This approach enhances patient care, reduces the need for additional procedures, and improves overall surgical outcomes [2-4].

Research and advancements in plastic surgery

It contributes to the advancement of plastic surgery by facilitating large-scale research studies, data sharing, and collaborations among surgeons and researchers. By analyzing aggregated data from multiple sources, data scientists can identify trends, patterns, and new insights that may lead to innovative surgical techniques, improved materials, and better patient care. This interdisciplinary collaboration between data scientists and plastic surgeons fosters continuous learning, knowledge exchange, and the development of evidence-based practices [4,5].

Ethical considerations and challenges

While data science offers numerous benefits in plastic surgery, it is essential to address ethical considerations and challenges associated with data privacy, consent, bias, and transparency. Protecting patient confidentiality, ensuring informed consent for data usage, addressing algorithmic biases, and maintaining transparency in data collection and analysis are crucial aspects to consider when integrating data science into plastic surgery practice [5].

Examples of various software

Several software applications have been utilized to enable surgeons with 3D models for a better level of evidence. Keep in mind that the field of technology and healthcare is constantly evolving, so there might be even more advanced tools available now. Here are a few examples that were practically being used at that time:

1. Advanced medical imaging software, such as OsiriX (Geneva, Switzerland: Pixmeo SARL), 3D Slicer (Boston, MA: The Surgical Planning Laboratory), and Mimics (Leuven, Belgium: Materialise NV), allows surgeons to create detailed 3D models from various medical imaging data like CT scans, MRIs, or ultrasounds. These 3D models provide a more comprehensive view of the patient's anatomy, aiding in preoperative planning, better understanding of complex structures, and improved decision-making during surgeries.

2. Virtual reality (VR) and augmented reality (AR) technologies have been adopted to enhance surgical training and planning. Software platforms like Touch Surgery (London, UK: Digital Surgery Ltd.), FundamentalVR (London, UK), and Precision OS (Vancouver, Canada) offer immersive experiences, allowing surgeons to practice procedures in virtual environments or overlay 3D models onto the patient during surgery. These applications provide valuable hands-on experience and spatial understanding to improve surgical outcomes.

3. Surgical navigation systems like Brainlab and Medtronic StealthStation (Dublin, Ireland: Medtronic plc) use 3D models to assist surgeons in real-time during procedures. By integrating preoperative imaging data with the actual surgical field, these systems offer precise guidance, aiding in minimally invasive surgeries and ensuring more accurate targeting of lesions or implants.

4. Custom implant design software are used in hand orthopedic and maxillofacial surgeries, custom implants are sometimes required to fit patients' unique anatomies. Software like Materialise Mimics inPrint (Leuven, Belgium: Materialise NV) and 3D-ME (Bentleigh East, Australia: Anatomics Pty Ltd) can generate personalized 3D models based on patient-specific data, allowing for the design and fabrication of patient-matched implants, resulting in better outcomes and faster recovery.

5. Various patient education software applications, such as ExplORer Surgical (Chicago, IL: ExplORer Surgical Inc.) and Touch Surgery, utilize interactive 3D models to help patients better understand their conditions and the proposed surgical procedures. Improved patient understanding can lead to increased compliance and potentially better post-operative outcomes.

6. Robotic surgery platforms like da Vinci Surgical System (Sunnyvale, CA: Intuitive Surgical, Inc.) use 3D visualization to provide surgeons with enhanced depth perception and better dexterity during minimally invasive procedures. These systems enable surgeons to perform complex operations with increased precision.

It's essential to note that while these software applications offer valuable support to surgeons and have the potential to improve surgical outcomes, they are meant to be complementary to the surgeon's expertise rather than a replacement for their skills and judgment. As technology continues to advance, the integration of 3D models and AI-driven tools is likely to become more prevalent in surgical practice. Always consult with healthcare professionals or up-to-date sources to get the latest information on software applications in the medical field.

In summary, data science has emerged as a powerful tool in the field of plastic surgery, revolutionizing patient selection, treatment planning, postoperative monitoring, and research. By harnessing the potential of data analytics, computational modeling, and machine learning, plastic surgeons can deliver safer, more precise, and personalized care to patients, ultimately shaping the future of the field and improving the lives of individuals seeking plastic surgery procedures.

## References

[1] Kanevsky J, Corban J, Gaster R, Kanevsky A, Lin S, Gilardino M (2016). Big data and machine learning in plastic surgery: a new frontier in surgical innovation. Plast Reconstr Surg.

[2] Ascha M, Ascha MS, Gatherwright J (2019). The importance of reproducibility in plastic surgery research. Plast Reconstr Surg.

[3] Borsting E, DeSimone R, Ascha M, Ascha M (2020). Applied deep learning in plastic surgery: classifying rhinoplasty with a mobile app. J Craniofac Surg.

[4] Thoma A, Murphy J, Voineskos SH, Coroneos CJ, Goldsmith CH (2022). Improving the science in plastic surgery. Plast Reconstr Surg.

[5] Cano SJ, Klassen A, Pusic AL (2009). The science behind quality-of-life measurement: a primer for plastic surgeons. Plast Reconstr Surg.

